# A Facile Aptasensor for Instantaneous Determination of Cadmium Ions Based on Fluorescence Amplification Effect of MOPS on FAM-Labeled Aptamer

**DOI:** 10.3390/bios11050133

**Published:** 2021-04-23

**Authors:** Yang Liu, Dongwei Zhang, Jina Ding, Kashif Hayat, Xijia Yang, Xuejia Zhan, Dan Zhang, Yitong Lu, Pei Zhou

**Affiliations:** 1 School of Agriculture and Biology, Shanghai Jiao Tong University, Shanghai 200240, China; kimi1201@sjtu.edu.cn (Y.L.); donaghy-zhang@sjtu.edu.cn (D.Z.); jnding@sjtu.edu.cn (J.D.); khayat97@sjtu.edu.cn (K.H.); eileenyang1986@sjtu.edu.cn (X.Y.); xjzhan@sjtu.edu.cn (X.Z.); zhdsjtu@sjtu.edu.cn (D.Z.); ytlu@sjtu.edu.cn (Y.L.); 2 Key Laboratory of Urban Agriculture, Ministry of Agriculture and Rural Affairs, Shanghai 200240, China; 3 Bor S. Luh Food Safety Research Center, Shanghai Jiao Tong University, Shanghai 200240, China

**Keywords:** aptasensor, cadmium, MOPS, fluorescence, FAM

## Abstract

Analytical performance and efficiency are two pivotal issues for developing an on-site and real-time aptasensor for cadmium (Cd^2+^) determination. However, suffering from redundant preparations, fabrications, and incubation, most of them fail to well satisfy the requirements. In this work, we found that fluorescence intensity of 6-carboxyfluorescein(FAM)-labeled aptamer (FAM-aptamer) could be remarkably amplified by 3-(N-morpholino)propane sulfonic acid (MOPS), then fell proportionally as Cd^2+^ concentration introduced. Importantly, the fluorescence variation occurred immediately after addition of Cd^2+^, and would keep stable for at least 60 min. Based on the discovery, a facile and ultra-efficient aptasensor for Cd^2+^ determination was successfully developed. The sensing mechanism was confirmed by fluorescence pattern, circular dichroism (CD) and intermolecular interaction related to p*K*_a_. Under the optimal conditions, Cd^2+^ could be determined rapidly from 5 to 4000 ng mL^−1^. The detection limit (1.92 ng mL^−1^) was also lower than the concentration limit for drinking water set by WHO and EPA (3 and 5 ng mL^−1^, respectively). More than a widely used buffer, MOPS was firstly revealed to have fluorescence amplification effect on FAM-aptamer upon a given context. Despite being sensitive to pH, this simple, high-performance and ultra-efficient aptasensor would be practical for on-site and real-time monitoring of Cd^2+^.

## 1. Introduction

Cadmium (Cd^2+^) is an important heavy metal and widely used in agriculture and industry [1]. However, this element is highly toxic, non-biodegradable, and bio-accumulative, which seriously threatens ecological security and human health [2]. It has been reported that continuous exposure to Cd^2+^ (even in trace concentration) may cause pathological disorders in entire human body system [3]. To this end, the regulatory guidance for Cd^2+^ in drinking water has been formulated by World Health Organization (WHO, 3 ng mL^−1^) and U.S. Environmental Protection Agency (EPA, 5 ng mL^−1^), respectively. In addition, various analytical techniques including electrothermal atomization atomic absorption spectroscopy (ET-AAS) [4,5,6], inductively coupled plasma mass spectroscopy (ICP-MS) [7,8,9,10] and graphite furnace atomic absorption spectrometry (GFAAS) [11,12], have been employed to determine Cd^2+^ in laboratory. As far as on-site and real-time determination and monitoring of Cd^2+^ in fields is concerned, the challenge comes as how to develop a convenient method without compromising performance and efficiency.

In recent decades, aptamer-based biosensors, i.e., aptasensors, have achieved substantial attention as robust detection tools. Aptamers are short nucleic acid oligomers selected by SELEX (systematic evolution of ligands by exponential enrichment) [13,14], which in turn enables them to recognize targets sensitively and selectively [15]. They are not only easy to synthesize, modify and store, but possess high affinity, low immunogenicity and toxicity [16]. By coupling those advantages with different signal output techniques, numerous aptasensors have been developed for sensitive detection of cadmium [17,18,19,20,21], lead [22,23,24,25,26], mercury [27,28,29,30], arsenic [31,32], and other heavy metals [33,34]. These aptasensors are accurate and sensitive, but most of them suffer from relatively poor efficiency. Banerjee reported a nanomaterial-based optical probe for sensitive determination of arsenic(III), where a low limit of detection (LOD) of 0.86 ppb was achieved. However, the synthesis and functionalization of the nanomaterial, Fe_3_O_4_(core)-Au(shell) nanocomposite, would consume more than 72 h. [35]. Similarly, relatively expensive instruments and toxic chemicals were required for the synthesis of TiO_2_-g-C_3_N_4_ before multistage fabrication of an impedimetric aptasensor [36]. In another case, the time cost for incubation even went up to 19 h for every measurement [19], where efficiency was inevitably lost. For the sake of fitting the aforementioned requirements for on-site and real-time monitoring, it is of paramount importance to develop a simpler and much more efficient aptasensor for Cd^2+^ determination.

**Scheme 1:**
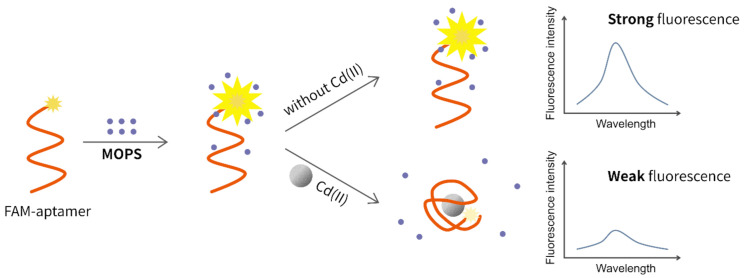
Schematic view of a facile and ultra-efficient aptasensor for instantaneous determination of Cd^2+^ based on fluorescence amplification effect of MOPS on FAM-aptamer.

In this work, we found that fluorescence intensity of FAM-aptamer could be remarkably enhanced by MOPS (a widely used buffer in bioassays), then dropped proportionally as a function of Cd^2+^ concentration introduced. Louder than that, the result obtained spoke the fluorescence variation would occur immediately after addition of Cd^2+^ into sensing system, and keep stable for at least 60 min. As the preparations for various nanoparticles and reagents, redundant procedures for fabrication and incubation were totally omitted, both performance and efficiency required for on-site and real-time determination of Cd^2+^ were satisfied perfectly. Based on the fresh discovery, a facile aptasensor for instantaneous determination of Cd^2+^ was developed and its schematic diagram was presented in Scheme 1.

## 2. Materials and Methods

### 2.1. Chemicals and Apparatus

The sequence of cadmium-specific aptamer (FAM-aptamer) was derived from its parent [21] by adding five nucleotides and labeling a FAM fluorophore at 3′ end: 5′-GGA CTG TTG TGG TAT TAT TTT TGG TTG TGC AGT CC-FAM−3′. The sequence was synthesized and purified by Sangon Biotech Co. (Shanghai, China). Standard solutions (1000 mg L^−1^) of Cd^2+^, Hg^2+^, and Pb^2+^ were purchased from Merck Co. (Germany). Other chemicals used in selectivity and recovery study, i.e., compounds with cationic of Ag^+^, Fe^3+^, Zn^2+^, As^5+^, As^3+^, Al^3+^, Ba^2+^, Sn^2+^, NH_4_^+^, Ca^2+^, Na^+^, and K^+^ were obtained from commercial sources and used without further purification. MOPS was obtained from Sangon Biotech Co. as well.

The fluorescence intensity was recorded by A Microplate Spectro-photometer M200 Pro (Tecan Group Ltd., Männedorf, Switzerland) at room temperature, with an excitation (emission) wavelength of 472 (520) nm and an excitation (emission) bandwidth of 9 (20) nm respectively. A J−1500 circular dichroism (CD) spectrometer (Jasco, Tokyo, Japan) was employed to characterize the steric configuration of FAM-aptamer, scanning from 190 to 300 nm with a bandwidth of 2 nm (200 nm min^−1^) three times. The response to MOPS was treated as baseline. All the ultrapure water utilized in this work was prepared by a Millipore-MilliQ system (Millipore Inc., Bedford, MA, USA).

### 2.2. Optimization of Experimental Conditions

Addition order for FAM-aptamer and Cd^2+^ was investigated. Firstly, 10 μL FAM-aptamer (20 nM, final concentration) was mixed into 230 μL MOPS (10 mM, pH 8.0), followed by addition of 10 μL Cd^2+^. After interaction, 200 μL from the resulting solution was transferred to a 96-well microplate for fluorescence measurement. The final concentrations of Cd^2+^ were set at two levels, a low level of 40 ng mL^−1^ and a high level of 200 ng mL^−1^. Similarly, assays with reversed addition order for FAM-aptamer and Cd^2+^ were then conducted as a contrast. According to the order of addition, they were denoted as MOPS + FAM-aptamer + Cd^2+^ and MOPS + Cd^2+^ + FAM-aptamer, respectively. Unless mentioned, all assays in this work were performed with a 250 μL sensing system, i.e., 10 μL aqueous sample containing Cd^2+^ and 10 μL FAM-aptamer mixed in 230 μL MOPS, then followed the same protocol for fluorescence measurement.

For optimization of concentration and pH of MOPS, a two-factor experiment with 5 levels (25 treatments in all) was operated. First, 50 mM MOPS was prepared and then diluted to 0.1, 1.0, 5.0, 10.0, and 20.0 mM with ultrapure water. Subsequently, the pH values of the resulting solutions were adjusted to 6.0, 7.0, 8.0, 9.0, and 10.0 by NaOH (1mM) respectively. Each treatment was marked as concentration * pH, e.g., 0.1 * 8.0 represented the treatment of 0.1 mM MOPS with a pH value of 8.0. Then within a 250 μL sensing system, the assays under different treatments of MOPS, as well as fluorescence measurement were performed in optimal addition order. The final concentrations of FAM-aptamer and Cd^2+^ were 20 nM and 200 ng mL^−1^, respectively.

To optimize incubation time, two experiments, i.e., (1) different concentrations of MOPS (1, 5, and 10 mM, pH 9.0) mixed with 20 nM FAM-aptamer and 200 ng mL^−1^ Cd^2+^ and (2) 5mM MOPS (pH 9.0) mixed with 20 nM FAM-aptamer and various concentrations of Cd^2+^ (10, 50, 100, and 200 ng mL^−1^), were respectively conducted at room temperature and completed by fluorescence measurement.

### 2.3. Sensitivity and Selectivity of the Sensing System

The sensitivity and selectivity of the sensing system were investigated under optimal conditions. Briefly, in sensitivity test, assays with increasing concentrations of Cd^2+^ (5–4000 ng mL^−1^) were performed. On the other hand, the selectivity was evaluated by the performance of sensing system under potential interferences including Pb^2+^, As^5+^, As^3+^, Ba^2+^, Ca^2+^, Al^3+^, Sn^2+^, NH_4_^+^, Na^+^, K^+^, Fe^2+^, and Fe^3+^.

### 2.4. Application in Water Samples

A recovery test was first implemented with ultrapure water spiked with multiple ions including Cd^2+^, Pb^2+^, Hg^2+^, As^5+^, As^3+^, Na^+^, K^+^, Al^3+^, Fe^2+^, Sn^2+^, and NH_4_^+^ with various concentrations. The recovery was calculated based on the spiked concentration of Cd^2+^ and the linear regression equation obtained in Section 2.3. No additional pretreatment was required before a measurement. Furthermore, freshwater samples from a tributary of Huangpu River were collected to assess the feasibility of the aptasensor against a complex matrix by another recovery test. The river water samples were firstly filtered through 0.22 µm membranes after spiking Cd^2+^, then measured following the protocol.

### 2.5. Calculation of Fluorescence Quenching and Fluorescence Quenching Efficiency

The fluorescence intensity of samples containing Cd^2+^ or interferences was recorded as *F*. *F*_0_ was assigned to the fluorescence intensity of a blank containing FAM-aptamer and MOPS only, where 10 μL aqueous sample containing Cd^2+^ was replaced by 10 μL ultrapure water. Fluorescence quenching (∆*F*) was defined as the difference between *F*_0_ and *F*, which can be calculated as ∆*F* = *F*_0_ − *F*; thus, fluorescence quenching efficiency (∆*F*/*F*_0_) can be calculated as ∆*F*/*F*_0_ = (*F*_0_ − *F*)/*F*_0_.

## 3. Results and Discussion

### 3.1. Sensing Mechanism

**Figure 1:**
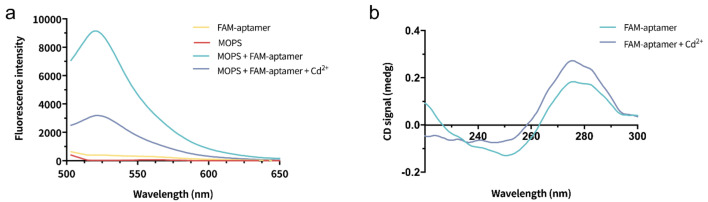
(**a**) Fluorescence emission spectra of FAM-aptamer, MOPS, MOPS + FAM-aptamer, and MOPS + FAM-aptamer + Cd^2+^ (FAM-aptamer, 20 nM; MOPS, 5mM, pH 9.0; Cd^2+^, 200 ng mL^−1^). (**b**) CD spectra of the FAM-aptamer (500 nM) in the absence and presence of 100 ng mL^−1^ Cd^2+^.

In current work, an interesting discovery that stood out was the fluorescence amplification effect of MOPS on FAM-aptamer, followed by a proportional decrease in fluorescence intensity as the concentration of Cd^2+^ added subsequently. As the results shown in Figure 1a, FAM-aptamer released feeble fluorescence with an intensity around 400, and MOPS showed almost no fluorescence in absence of FAM-aptamer and Cd^2+^. However, a significantly stronger fluorescence could be recorded when FAM-aptamer and MOPS were mixed. The fluorescence intensity was enhanced by more than 22 times and mounted up beyond 9000. No previous study was reported on this phenomenon. We speculated that there were probably two reasons. On one hand, it was obvious that the 3′ end of FAM-aptamer was complementary to its 5′ end. The FAM-aptamer in random coil status might be inclined to self-stack by base pairing without buffer, enabling chances to weaken and even screen the fluorescence to a certain extent. On the other hand, MOPS also offered microenvironment for protonation and deprotonation while dispersing FAM-aptamer homogeneously as a buffer. The resulting charged microspecies of FAM interacted with deprotonated MOPS through electrostatic force and hydrogen bond (mainly resulting from N and O atoms), enabling energy transfer induced by electric dipoles and then fluorescence enhancement. Afterwards, with addition of 200 ng mL^−1^ Cd^2+^, the fluorescence of system dropped dramatically to around 3000. This could be ascribed to the specific recognition of FAM-aptamer to Cd^2+^. Since Cd^2+^ was added in the aforementioned system, the FAM-aptamer disengaged themselves from the interaction with MOPS and was induced to form a stem-loop structure (partial hybridization by six base-pairs) to bind Cd^2+^ with higher affinity simultaneously [37]. This formation prevented MOPS from approaching FAM fluorophore by steric effect, leading to reduced energy transfer and weak fluorescence. This resulting fluorescence pattern demonstrated the feasibility of the proposed method. Based on the fluorescence variation proportional to Cd^2+^ concentration added, a facile and sensitive aptasensor for Cd^2+^ determination was successfully developed. The schematic was displayed in Scheme 1. MOPS has been reported to own surfactant-like effect [38,39] and be able to inhibit catalytic activity [40]. The fresh discovery in current work, that was fluorescence amplification effect on FAM-aptamer, further advanced our knowledge of MOPS more than a widely used buffer.

Then, CD measurements were applied to investigate the conformational alteration of FAM-aptamer in presence of Cd^2+^. As shown in Figure 1b, the FAM-aptamer presented a negative peak around 250 nm and a positive peak at 275 nm, which was in accordance with the spectra of previous study [21]. After introduction of 100 ng mL^−1^ Cd^2+^, the negative peak decreased while an enhanced positive peak around 275 nm was observed, indicating the binding of FAM-aptamer to Cd^2+^ through conformation change.

### 3.2. Optimization of Addition Order

There were two interactions within this sensing system, i.e., the specific recognition of FAM-aptamer to Cd^2+^ and fluorescent amplification of MOPS on FAM-aptamer. As FAM-aptamer was involved in both two interactions, the optimization of addition order was of significance.

**Figure 2:**
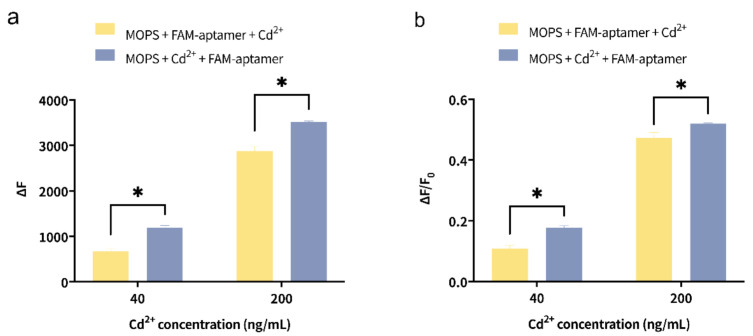
Effect of addition order on (**a**) fluorescence quenching (∆*F*) and on (**b**) fluorescence quenching efficiency (∆*F*/*F*_0_). * indicates significant difference (*p* < 0.05, n ≥ 3).

As the results shown in Figure 2, ∆*F* and ∆*F*/*F*_0_ of MOPS + Cd^2+^ + FAM-aptamer were significantly higher than that of MOPS + FAM-aptamer + Cd^2+^ (*p* < 0.05) upon both low and high concentration of Cd^2+^. This could be explained as follows. When FAM-aptamer was firstly mixed into MOPS, they dispersed homogeneously at the very beginning, followed by interaction to initiate energy transfer. Therefore, prior to binding Cd^2+^ added subsequently, they needed to disengage themselves from the interaction. In other words, the interaction between FAM-aptamer and MOPS retarded the specific recognition of FAM-aptamer to Cd^2+^ in the addition order of MOPS + FAM-aptamer + Cd^2+^. However, there was insufficient evidence to support the interaction between MOPS and Cd^2+^ by now. Due to higher affinity to Cd^2+^, it would be easier for FAM-aptamer to bind Cd^2+^ if Cd^2+^ was added before FAM-aptamer. Hence the addition order of MOPS + Cd^2+^ + FAM-aptamer was adopted in following assays.

### 3.3. Optimization of Concentration and pH of MOPS

**Figure 3:**
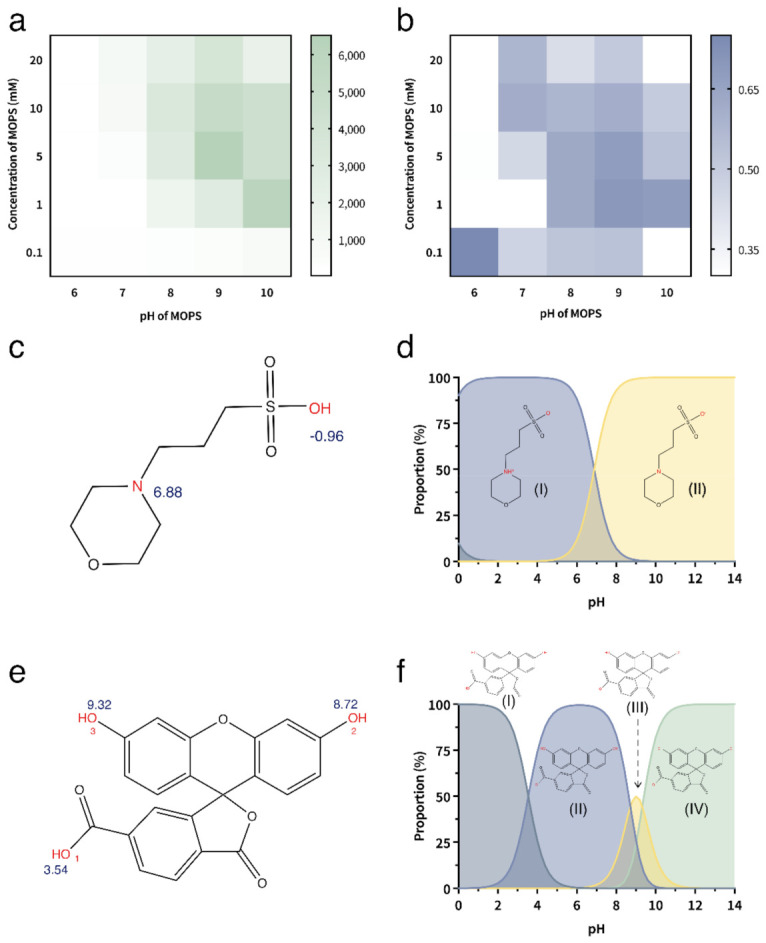
Interactive effect of concentration and pH of MOPS on (**a**) fluorescence quenching (∆*F*) and (**b**) fluorescence quenching efficiency (∆*F*/*F*_0_). (**c**) MOPS molecule with p*K*_a_ values of O and N sites and (**d**) distribution of its major charged microspecies upon varied pH. (**e**) FAM fluorophore molecule with p*K*_a_ values of O sites and (**f**) distribution of its charged microspecies upon varied pH.

As a key component to the sensing system, the effects of concentration and pH of MOPS on fluorescence quenching were simultaneously factored into overall investigation, and then plotted as two heatmaps (Figure 3a,b). For this assay, the pH of MOPS seemed to be more significant. On one hand, FAM was reported to be pH sensitive [41]. On the other hand, the pH directly determined the degree of protonation and deprotonation, then influenced energy transfer. Therefore, to further understand the effect, the p*K*_a_ values of both FAM and MOPS were involved and evaluated with Marvin Sketch software (Figure 3c,e) [42].

As shown in Figure 3a, ∆*F* was mostly less than 500 when pH ≤ 7, which could be explained from the aspect of p*K*_a_ and pH. Figure 3c indicated the p*K*_a_ of O and N site in MOPS was ~−0.96 and ~6.88, respectively. When pH ≤ 7, autoprotolysis of MOPS dominated and the resulting microspecies (I) (Figure 3d) was simultaneously positively charged on N site and negatively charged on O site. The strong intermolecular electrostatic adsorption led to self-aggregation of MOPS and less interaction with FAM fluorophore. Although a treatment like 0.1*6 presented excellent performance in ∆*F*/*F*_0_ in this area, its low ∆*F* was easily distracted by background noise or interferences, then failed to determine Cd^2+^ accurately. While pH lay in the range from 8 to 10, the ∆*F*/*F*_0_ increased firstly and then decreased as increasing concentration of MOPS at a certain pH. Upon all the treatments here, the treatment of 5.0*9.0 stood out with the highest ∆*F* and a ∆*F*/*F*_0_ exceeding 0.67 (Figure 3a,b). As shown in Figure 3d,f, the majority of MOPS deprotonated to microspecies (II) while three different deprotonated forms of FAM coexisted at this stage. This phenomenon demonstrated that fluorescence variation of the electric dipole-induced energy transfer was mainly affected by microspecies (II) of MOPS and microspecies (III) of FAM, the most abundant deprotonated form at pH 9.0. We reasoned the energy transfer intensity was like a game of electrostatic force, hydrogen bond, and steric effect. The stronger the hydrogen bond, the tighter the adsorption, accompanying by more intense steric effect and electrostatic repulsion. The appropriate deprotonation degree of three O sites in microspecies (III) of FAM could tune this game to the best performance in energy transfer. Finally, given that the formation of cadmium hydroxyl might confuse the determination of Cd^2+^, the treatments upon pH > 10 were ruled out [43]. In summary, 5 mM MOPS with pH 9.0 was selected and used in following assays.

### 3.4. Optimization of Incubation Time

**Figure 4:**
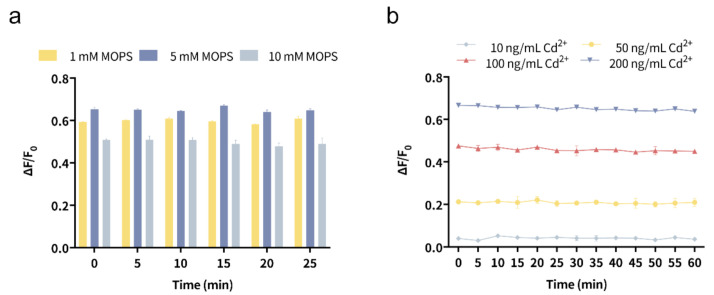
Stability of fluorescence quenching efficiency (∆*F*/*F*_0_) in time scale under (**a**) different concentrations of MOPS (1, 5, and 10 mM, pH 9.0) mixed with 20 nM FAM-aptamer and 200 ng mL^−1^ Cd^2+^, and under (**b**) 5mM MOPS (pH 9.0) mixed with 20 nM FAM-aptamer and various concentrations of Cd^2+^(10, 50, 100, and 200 ng mL^−1^) at room temperature. (n ≥ 3).

In order to achieve the best performance and efficiency of the proposed aptasensor, experiments were conducted to optimize incubation time. The results obtained were summarized in Figure 4. First of all, the highest ∆*F*/*F*_0_ value under the treatment of 5 mM MOPS (pH 9.0) further supported the conclusion in Section 3.3 (Figure 4a). However, the most striking finding was ∆*F*/*F*_0_ value almost remained constant from the very beginning and would keep stable for 60 min at least (Figure 4b). These results demonstrated the remarkable long-term stability of the aptasensor. Louder than that, it spoke that the high-performance sensing could be instantly accomplished, suggesting the aforementioned goal of on-site and real-time determination could be well achieved with this robust aptasensor.

### 3.5. Sensitivity

**Figure 5:**
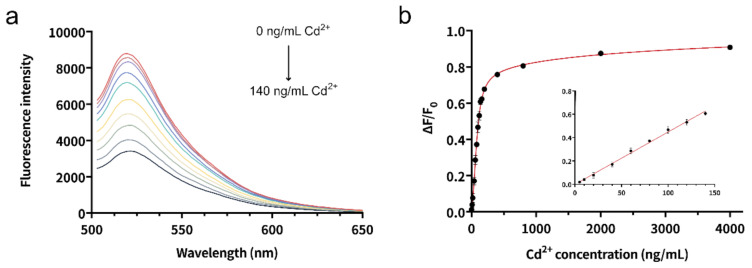
(**a**) Fluorescence emission spectra of the aptasensor on exposure to 0, 5, 10, 20, 40, 60, 80, 100, 120, and 140 ng mL^−1^ Cd^2+^. (**b**) Calibration curves of the aptasensor for Cd^2+^ determination. (Insert) Initial linear responses in the range of 5−140 ng mL^−1^ Cd^2+^. (n ≥ 4).

Under the optimal experimental conditions, the analytical performance of the aptasensor was investigated. At first, the sensitivity was evaluated by adding increasing concentrations of Cd^2+^ into sensing system. As depicted in Figure 5a, the fluorescence intensities of the aptasensor decreased gradually as a function of Cd^2+^ concentrations added. The ∆*F*/*F*_0_ was plotted against Cd^2+^ concentration from 5 to 4000 ng mL^−1^ (Figure 5b), and the inset presented a good linear relationship upon the concentration range of 5 to 140 ng mL^−1^. The obtained linear regression equation was shown below:

∆*F*/*F*_0_ = 0.0045*C* − 0.0015 (*R*^2^ = 0.996)

*C*: Cd^2+^ concentration; *R*^2^: correlation coefficient.

The LOD was estimated as 1.92 ng mL^−1^ (3σ/slope), which was lower than the maximum concentration limit set by WHO (3 ng mL^−1^) and EPA (5 ng mL^−1^), respectively. Moreover, a comparison of existing aptamer-based methods for Cd^2+^ determination was conducted (Table 1). The table showed that in most cases, efficiency was severely limited by incubation beyond hours, even if time cost for preparations of nanoparticles and multistage fabrication procedures were not yet included. With current aptasensor, however, incubation was needless, curtailing the total time cost dramatically. Given its simplicity and near-perfect performance in efficiency, a comparable LOD with other approaches was acceptable. These results not only revealed the superior efficiency of the aptasensor, but also demonstrated its promising future for real-time monitoring of Cd^2+^ in fields.

### 3.6. Selectivity

**Figure 6:**
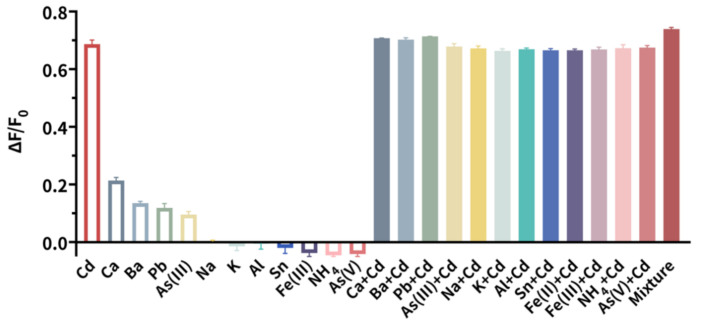
Selectivity of the aptasensor towards other potential interfering ions in absence and presence of Cd^2+^. The concentration of Cd^2+^ was 200 ng mL^−1^, and that of all other ions was 400 ng mL^−1^. (n ≥ 3).

The selectivity of the method was evaluated by recording the response of the aptasensor towards other potential interfering ions in absence and presence of Cd^2+^, respectively. Due to high affinity to Cd^2+^, the other ions could hardly induce the disengagement of FAM-aptamer from MOPS despite their excessively high concentrations. Figure 6 showed all other ions had ∆*F*/*F*_0_ lower than 0.2, while the ∆*F*/*F*_0_ for Cd^2+^ approached 0.7. After the introduction of Cd^2+^ to the above individual ion sensing system and the mixture, the sharply enhanced ∆*F*/*F*_0_ confirmed the high selectivity of the aptasensor for Cd^2+^.

Furthermore, it was reported that K^+^, Na^+^, and Pb^2+^ could induce a G-rich ssDNA to form a structure of G-quartet (or G-quadruplex), then significantly quenched the fluorescence of fluorophore [51]. The significant differences here basically neutralized the concerns about G-quadruplex, as well as the concerns about distraction of metal ions-directed fluorescence quenching [52].

### 3.7. Reproducibility

As the merits of simplicity and robustness that the system possessed, the reproducibility was well guaranteed by nature. It was evaluated by 10 assays within 200 ng mL^−1^ Cd^2+^ under identical conditions. The ∆*F*/*F*_0_ value obtained with a relative standard deviation (RSD) of 0.74% totally eliminated the concerns over reproducibility.

### 3.8. Determination of Cd^2+^ in Water Samples

The feasibility of the current aptasensor was evaluated by Cd^2+^ determination in several spiked ultrapure water samples containing various concentrations of Cd^2+^ and other interfering ions. As the results exhibited below (Table 2), the recoveries with the artificial samples ranged from 96.79% to 105.22% with RSD values of 0.64% to 3.12%, indicating its good analytical performance against interfering ions.

Environmental samples were generally more complicated, where a variety of both organic and inorganic interferences beyond the aforementioned ions existed. To further assess the practicability of the aptasensor against a complex matrix, freshwater samples from a tributary of Huangpu River were collected for another recovery test. While the fluorescence signal was slightly distracted by various interferences in river water, the recoveries ranging from 91.29% to 107.74% with RSD values of 4.80% to 7.94% was still good and acceptable (Table 3).

The results obtained here with artificial and environmental water samples not only demonstrated robustness in anti-interference of the aptasensor, but also substantiated its promising application in fields.

## 4. Conclusions

In summary, a facile aptasensor for instantaneous determination of Cd^2+^ was developed based on the fluorescence amplification effect of MOPS on FAM-aptamer. Under the optimal conditions, Cd^2+^ could be determined from 5 to 4000 ng mL^−1^ in a matter of seconds. The detection limit of 1.92 ng mL^−1^ was significantly lower than the maximum concentration limit for drinking water set by WHO (3 ng mL^−1^) and EPA (5 ng mL^−1^). The primary advantages of the aptasensor lay in its ultra-efficiency and simplicity. Since unnecessary preparation of nanoparticles, redundant fabrication procedures, and incubation time were totally omitted, the execution efficiency (both at lab and in field) had been pushed to a new level by current system, demonstrating its great potential for real-time and on-site applicability. Interestingly, MOPS was reported to have fluorescence amplification effect on FAM-aptamer in a given context, advancing our knowledge of MOPS more than a widely used buffer. Although the system possessed the aforementioned merits, there were still some limitations existing. The performance was sensitive to pH, of which should be taken care. Its availability could be further improved if integration into a portable device was realized. Lastly, instead of “signal-off” strategy, a “signal-on” aptasensor would be more favorable, where the signal increased with the increasing concentration of target. Continuous endeavors would be made to push that development.

## Figures and Tables

**Table 1:** Comparison of different aptamer-based approaches for Cd^2+^ determination.

Approaches	Linear Range (ng mL^−1^)	LOD (ng mL^−1^)	Incubation Time	Reference
Colorimetry	1.12–44.8	0.52	65 min	[21]
	1–400	1	80 min	[44]
	0.056–22.48, 33.72–112.41	0.017	105 min	[45]
	1.12 × 10^−3^–112	1.12 × 10^−3^	>150 min	[46]
	5–100	2.4	50 min	[47]
Electrochemistry	0.1–1000	0.05	>45 min	[48]
	28–112	10.3	>19 h	[19]
Glucometer	2.24 × 10^−3^–22.4	5.6 × 10^−4^	180 min	[17]
Fluorometry	0–112	4.48	30 min	[20]
	100–10^5^	0.038	20 min	[49]
	0.81–22.4, 22.4–560	0.24	30 min	[18]
	1.12–224.82	0.34	70 min	[50]
	5–140	1.92	<1 min	This work

**Table 2:** Determination of Cd^2+^ in artificial water samples (n ≥ 3).

Spiked (ng mL^−1^)	Interfering Ions (ng mL^−1^)	Found (ng mL^−1^)	Recovery (%)	RSD (%)
10	Pb2+ (10), Hg2+ (10), As5+ (10), As3+ (10), Na+ (10), K+ (10), Al3+ (10), Fe2+ (10), Sn2+ (10), NH4+ (10)	9.68	96.79	2.36
50	Hg2+ (50), As5+ (50), As3+ (50), Na+ (50), K+ (50), Al3+ (50), Fe2+ (50), Sn2+ (50), NH4+ (50)	48.43	96.85	3.12
100	Pb2+ (100), As5+ (100), As3+ (100), Na+ (100), K+ (100), Al3+ (100), Fe2+(100), Sn2+ (100), NH4+ (100)	105.22	105.22	0.64

Note: the numbers in parentheses after each ion show the final concentration of the ions added.

**Table 3:** Determination of Cd^2+^ in environmental water samples (n ≥ 3)

Background (ng mL^−1^)	Spiked (ng mL^−1^)	Found (ng mL^−1^)	Recovery (%)	RSD (%)
0.02	10	9.15	91.29	7.94
	50	53.89	107.74	4.80
	100	97.55	97.53	5.07

## Data Availability

Not applicable.
